# Pharmacokinetics of two pharmaceutical presentations of benznidazole in adult *Trypanosoma cruzi* infected population

**DOI:** 10.1590/0074-02760240177

**Published:** 2025-02-28

**Authors:** Yolanda Hernández, María Elena Marson, Marisa Liliana Fernández, Omar Sued, Claudia Frola, Santiago Perez Lloret, Pedro Cahn, Nilda Graciela Prado, Guido Enrique Mastrantonio Garrido, Sergio Sosa-Estani

**Affiliations:** 1 Administración Nacional de Laboratorios e Institutos de Salud Dr Carlos G Malbrán, Instituto Nacional de Parasitología Fatala Chaben, Ciudad Autónoma de Buenos Aires, Argentina; 2 Universidad Nacional de La Plata, Facultad de Ciencias Exactas, La Plata, Provincia de Buenos Aires, Argentina; 3 Consejo Nacional de Investigaciones Científicas y Técnicas, Argentina; 4 Unidad Planta Laboratorio, Comisión de Investigaciones Científicas de la Provincia de Buenos Aires, Argentina; 5 Fundación Huésped, Ciudad Autónoma de Buenos Aires, Argentina; 6 Pontificia Universidad Católica Argentina, Observatorio de Salud, Ciudad Autónoma de Buenos Aires, Argentina

**Keywords:** pharmacokinetics, benznidazole, pharmaceutical presentations, chronic infection

## Abstract

**BACKGROUND:**

Benznidazole (BNZ) is the primary treatment for Chagas disease. While pharmacokinetic studies of BNZ began in the 1970s, its metabolism and excretion are not fully understood. Alternatives like Benznidazol Lafepe^®^ and Abarax^®^ have replaced the original Radanil^®^.

**OBJECTIVES:**

To compare the pharmacokinetic profiles of both currently available formulations of BNZ in adults with chronic *Trypanosoma cruzi* infection.

**METHODS:**

The study involved 13 subjects each one receiving 100 mg of both presentations one week apart. Blood samples were collected over 48 hours post-administration to analyse BNZ concentration and calculate pharmacokinetic parameters.

**FINDINGS:**

The analysis showed that both presentations had similar maximum plasma concentration and time to reach maximum plasma concentration values. Area under curve (AUC) values were slightly lower in Abarax^®^ than Benznidazol Lafepe^®^. High intra-individual variability was observed, attributed to erratic absorption patterns with multiple peaks in concentration-time curves. The half-life values for both formulations were 9.1 and 8.0 h, respectively, with a significant intra-individual variability over 30%.

**MAIN CONCLUSIONS:**

The mean difference in the AUC was lower than 10%, but exceeded the 90% confidence interval for the higher bioequivalence limit. Despite the high variability that confirms erratic absorption, the pharmacokinetic parameters of both formulations were within expected ranges.

Chagas disease (CD) is a systemic parasitic infection caused by *Trypanosoma cruzi*. It is considered a neglected disease,^1^ being endemic in 21 countries in the Americas and, due to migration phenomena, it has spread to non-endemic countries in Asia, Oceania and Europe.^2^ ^,^ ^3^ According to 2010 data, it is estimated that more than 5.7 million people were infected with *T. cruzi* and it is the cause of about 12,000 deaths annually.^4^

CD is characterised by an acute phase, generally oligosymptomatic. Once the infection initiates, the progression to a chronic phase takes two months. This phase is, in most patients (60-70%) asymptomatic, with no proven pathology (indeterminate). However, up to 30-40% of patients could develop cardiac and/or digestive pathology after 10-30 years of the primary infection.^1^ ^,^ ^5^ ^,^ ^6^

The only drugs with trypanocidal action approved for treatment in humans are a nitroimidazole, benznidazole (BNZ), and a nitrofuran, nifurtimox, both of which have been available for this purpose for over 50 years, although for various reasons BNZ is the treatment of first choice. The recommended dose for BNZ treatment in adult patients is 5-7 mg/kg/day orally in two doses for 30 to 60 days. Trypanocidal treatment has been shown to be effective in the acute phase of the disease, with cure rates of approximately 65-80%, reaching almost 100% in cases of congenital infection treated within the first year of life.^1^ ^,^ ^5^ It has also been shown to be effective in recent chronic infection such as in children aged 5-12 years, as demonstrated in two randomised clinical trials^6^ with less effectiveness in the chronic phase in adults.^7^ ^-^ ^13^

Clinical use of BNZ in CD has consistently demonstrated that it has a relevant toxicity profile, with the severity of adverse events leading to definitive discontinuation of trypanocidal treatment in up to 30% of treated adult patients.^1^ ^,^ ^14^ This rate is much lower in paediatric patients, with mostly mild manifestations that rarely justify discontinuation of treatment.^15^

The first studies determining pharmacokinetic parameters in humans for BNZ were conducted by Raaflaub and Ziegler in 1979 and 1980, respectively. The first consisted of a single-dose design in which a 100 mg BNZ tablet was administered to six healthy volunteers.^16^ In the second one used a multiple-dose design, where eight adult patients with CD were included and a dose of 7 mg/kg/day was administered.^17^ Both studies used the formulation Radanil^®^ (Roche Laboratories).

Faced with an uncertain scenario in many aspects of pharmacotherapy, pharmacokinetic studies in humans were resumed several years later,^18^ ^,^ ^19^ ^,^ ^20^ ^,^ ^21^ ^,^ ^22^ which still do not provide an absolutely clear picture of the behaviour of BNZ in patients, under different conditions and principally for the different available formulations. Recently, consensus pharmacokinetic parameters have been proposed, based on information available in the literature,^23^ which in this work are contrasted in a comparative study for two of the available solid formulations, Benznidazol Lafepe^®^ (Laboratório Farmacêutico do Estado de Pernambuco Governador Miguel Arraes, Brazil) and Abarax^®^ (Laboratorios Elea, Argentina).

In the context of its attributes as a neglected disease, it must be considered that information regarding the pharmacokinetics, metabolism, excretion and pharmacodynamics of BNZ is still incomplete. Current pharmacological protocols should be revised in order to improve the efficacy and to reduce adverse effects. For example, adjusting doses in the paediatric therapeutic^18^ and modifying schemes (daily dose and treatment duration) in the adult therapeutic.^24^ ^,^ ^25^ These alternatives, together with the search for new formulations of the same drugs and combined pharmacotherapies, are the most promising lines of research into new treatments.^26^ ^,^ ^27^

An important aspect to consider is that the original formulation of BNZ (Radanil^®^) has not been on the market since 2011. Benznidazol Lafepe^®^ and, since 2012, the formulation Abarax^®^ are both available, being Abarax^®^ currently the most word widely used pharmaceutical form for the treatment of infected patients. Also, Abarax^®^ was approved by the United States Food and Drug Administration (FDA) in 2017 for its use in children. Since the information on the pharmacokinetic phenomena of BNZ is incomplete, complementary studies are necessary. Knowledge gaps in the pharmacokinetics of BNZ are related to the influence of formulations, drug-drug interaction and the effect of individual metabolic parameters, among others.^28^

This study describes a comparison of the pharmacokinetic profile of two pharmaceutical presentations (Abarax^®^ and Benznidazol Lafepe^®^), administered as single doses in a cohort of adults infected with *T. cruzi*, in the chronic phase of the disease, with no proven pathology. Both formulations tested in the present study belong to class III of the biopharmaceutical classification (BCS), characterised by rapid dissolution of the pharmaceutical formulation and slow intestinal absorption. This study does not qualify as a bioequivalence study, as it fails to comply with all of the requirements stipulated by Argentine regulations (ANMAT Disposition Nº 3185/1999). However, to the best of our knowledge, this is the first comparative pharmacokinetic study between available solid formulations of BNZ.

## SUBJECTS AND METHODS

*Study population* - Thirteen volunteers were selected and included by the Clinical, Pathology and Treatment Department of the Dr Mario Fatala Chaben National Institute of Parasitology (Buenos Aires, Argentina), between June 2014 and April 2015. This cohort underwent pharmacokinetic study procedures at Fundación Huésped (Buenos Aires, Argentina).

Inclusion criteria considered subjects residing in Buenos Aires, aged between 21 and 50 years, with body mass index (BMI) between 19 and 29 kg/m². All had reactive serology for *T. cruzi* considering those with positive results for at least two of three different serological techniques (indirect immunofluorescence, indirect haemagglutination and enzyme-linked immunosorbent assay - ELISA), without Chagas pathology (indeterminate), demonstrated by the absence of cardiac and gastrointestinal alterations.

Exclusion criteria were a history of drug or alcohol abuse in the last two years, smokers, intake of other drugs in the two weeks prior to the trial, history of gastrointestinal surgery, patients with digestive or metabolic pathologies that alter absorption, history of cardiovascular, hepatic, renal, haematological, metabolic, bladder, psychiatric or other diseases considered unsuitable for participation in the study, abnormal electrocardiogram and chest X-ray, impaired hepatic or renal function, blood donation or participation in another study within the last three months, reactive serological tests for human immunodeficiency virus (HIV), hepatitis A (Ig M), hepatitis B (HbsAg), hepatitis C (anti-HCV), syphilis (VDRL), positive urine drug of abuse and/or alcohol, laboratory and/or clinical examination results with clinically significant abnormalities, volunteers who are to start scheduled medical or pharmacological treatment outside this protocol expressing risk of non-adherence to the protocol, and women of childbearing age with a positive serum pregnancy test or breastfeeding. Additional exclusion criteria were known hypersensitivity to BNZ or any of the excipients in the formulations which are corn starch, lactose hydrate, croscarmellose sodium, magnesium stearate and microcrystalline cellulose PH 102.

*Study implementation* - A Phase IV bioavailability study with Phase I methodology, open to patients and clinical investigators, blinded to bioanalytical investigators, non-randomised, non-replicated and balanced, with two treatments, with single doses in each was carried out. All subjects received, on an empty stomach to minimise possible postprandial effect, one 100 mg tablet of Abarax^®^ and one 100 mg tablet of Benznidazol Lafepe^®^ with an interval of one week between intakes, with 12 h of hospitalisation for each treatment, with a sample collection period of 48 h and a rest period of five days between both treatments.

Subjects received a standardised meal during hospitalisation, medical and nursing monitoring, and a safety laboratory was performed at the end of the study.

*Sampling and analysis* - In each subject and collection series, 6 mL of blood was obtained with a heparinised syringe at different times. Samples were obtained prior to drug administration (0 h) and close to 0.5; 1.0; 2.0; 2.5; 3.0; 3.5; 4.0; 4.5; 6.0; 8.0; 12; 24 and 48 h after the drug administration, being exactly recorded the sampling time. Each sample was placed in a polyethylene tube, centrifuged and then fractionated into three 1,000 mL equivalent aliquots of plasma. Plasma aliquots were placed in cryogenic tubes with hermetic screw caps and immediately freezed (-20ºC), prior to its transport and analysis at the Unidad Planta Laboratorio (UPL) of the Comisión de Investigaciones Científicas de la Provincia de Buenos Aires (La Plata, Argentina).

Two equivalent aliquots of each serum sample were processed independently, reserving a third aliquot for repetition in case of discordance of the first two aliquots, as established in the previously reported method.^29^ Briefly, we first proceeded to lyophilisation and then, 2.0 mL of ethyl acetate and 100 µL of trichloroacetic acid (30% w/v aqueous solution) were subsequently added, followed by shaking and sonication for 6 min. The mixture was centrifuged at 8000 g for 10 min. The supernatant was brought to dryness under vacuum (40ºC and 90 - 120 bar) and the residue was resuspended in 600 µL of buffer solution pH 2.4 glycine/acetonitrile. The analysis was performed by high performance liquid chromatography (HPLC) using a standard C18 column, with isocratic elution (glycine/acetonitrile buffer) and injection of 20 µL, in duplicate and detection by UV spectrometry at 313 nm, quantified by external calibration duplicate curve. A total of 16 batches were measured, with an intra-assay control sample (15 µg/mL) in each batch and two calibration series, at the beginning of each working week. The methodologies and equipment were implemented under conditions of certifiability and application of Good Laboratory Practices, according to ANMAT Disposition Nº5040/06.

*Pharmacokinetic analysis* - Pharmacokinetic analysis was performed according to protocol. The non-compartmental parameters of area under the curve from time zero to time t (AUC_0-t_) and from time zero to infinity (AUC_0-∞_) were calculated using the trapezoid method. C_max_ was the maximum plasma concentration after drug intake and T_max_ was the time at which this value occurred. The elimination constant (Ke) and half-life (t_½_) were calculated by regression analysis, including at least four points.

For the statistical analysis, a mixed-effects analysis of variance (ANOVA) was used, including only the inter-subject factor “treatment” and one intra-subject factor. AUC_0-t_, AUC_0-∞_ and C_max_ values were analysed. The statistical analysis was performed considering that the assignment of the initial tablet in each subject was without randomisation.

*Ethics* - All patients signed the informed consent form and the protocol was approved by the Bioethics Committee of Fundación Huésped.

## RESULTS

The subjects who entered the study were nine females and four males, for whom the mean height was 161.3 ± 8.6 cm, with a mean weight of 63.5 ± 10.1 kg. Mean age (38.7 ± 9.3 years) and BMI (24.3 ± 2.3 kg/m²) were within the range determined by the inclusion criteria. The mean values (pre- and post-trial) of plasma clinical parameters are shown in Table I, with no significant differences between trials. No serious adverse events were observed during the conduct of the trials. However, two patients showed manifestations associated with concomitant pathologies and two patients had hematomas at the venipuncture site.

**TABLE I:** Comparison of plasma parameters of patients before and after the study

Parameter	Pre-study	Post-study
Age patient (year)	38.7 ± 9.3	-
Weight patient (Kg)	63.5 ± 10.1	-
Height patient (cm)	161.3 ± 8.6	-
Body mass index patient (Kg/m²)	24.3 ± 2.3	-
Blood haemoglobin (gr/dL)	13.6 ± 1.5	13.0 ± 1.5
Blood leukocytes (cells/µL)	6423 ± 1161	5992 ± 1360
Blood platelets (cells/µL)	237923 ± 42506	230154 ± 46560
Seric creatinine (mg/dL)	0.9 ± 0.1	0.8 ± 0.1
Uremia (mg/dL)	30.5 ± 5.9	31.1 ± 7.1
GOT (U/L)	20.5 ±10.2	22.1 ± 8.8
GPT (U/L)	26.5 ± 27.9	30 ± 29.9

GOT: glutamic oxaloacetic transaminase seric activity; GPT: glutamic pyruvic transaminase seric activity.

The bioanalytical calibration yielded a limit of quantification (LOQ) of 0.32 µg/mL, a limit of detection (LOD) of 0.14 µg/mL, a sensitivity of 0.04 µg/mL, a recovery of 91% (at 1.00 µg/mL level) and a median percentage coefficient of variation of 8.0%. The percent coefficient of variation of the control series was 4.9%, giving an average value of 14.96 µg/mL, being both values optimal as bioanalytical validation parameters.

**Fig. 1:**
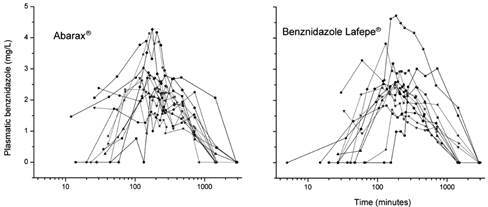
plasma concentration of benznidazole (mg/mL) relative to intake time (minutes) following administration of Abarax^®^ or Benznidazol Lafepe^®^ for individual measures.

The Fig. 1 shows the log-scale concentration-time curves of the 13 patients of the trial with each formulation. Table II shows the comparisons of pharmacokinetic parameters between treatments with both formulations. Table III shows the comparison between the bioavailability of both formulations. The 90% confidence interval (CI) for C_max_ was within the pre-specified 80-125% range, while for AUC, the upper end of the CI exceeded the pre-specified limit, giving an intra-individual variability of 40.7% for the same parameter.

**TABLE II:** Pharmacokinetic parameters for Abarax^®^ (A) and Benznidazol Lafepe^®^ (B)

		A (n = 13)	B (n = 13)
C_max_ (µg/mL)	Media ± SD	3.0 ± 0.8	2.9 ± 0.8
	CI 95%	2.5 - 3.4	2.5 - 3.3
	Min-Max	1.8 - 4.3	1.7 - 4.7
T_max_ (h)	Media ± SD	3.2 ± 1.6	3.5 ± 2.7
	CI 95%	2.4 - 4.1	2.1 - 5.0
	Min-Max	2.0 - 8.0	1.0 - 12.0
AUC_0-t_ (µg·h/mL)	Media ± SD	32.0 ± 9.3	37.5 ± 21.1
	CI 95%	27.0 - 37.1	26.1 - 49.0
	Min-Max	15.8 - 56.3	15.8 - 84.4
AUC_0-∞_ (µg·h/mL)	Media ± SD	32.0 ± 9.3	37.5 ± 21.1
	CI 95%	27.0 - 37.1	26.1 - 49.0
	Min-Max	15.8 - 56.3	15.8 - 84.4
Ke (h^­^¹)	Media ± SD	-0.1 ± 0.1	-0.1 ± 0.0
	CI 95%	-0.1- -0.1	-0.1- -0.1
	Min-Max	-0.2 - 0.01	-0.2 - 0.01
t_1/2_ (h)	Media ± SD	8.0 ± 3.5	9.1 ± 3.6
	CI 95%	6.1 - 9.9	7.1 - 11.0
	Min-Max	3.4 - 13.5	3.9 - 14.5
MRT_0-∞_	Media ± SD	9.1 ± 3.5	9.5 ± 3.1
	CI 95%	7.2 - 11.0	7.8 - 11.2
	Min-Max	5.1 - 17.6	5.7 - 14.4

AUC_0-t_: area under curve from zero to 48 h; AUC_0-∞_: area under curve from zero to infinity; CI: confidence interval; C_max_: maximum plasma concentration after benznidazole intake; Ke: benznidazole elimination constant; MRT_0-∞_: benznidazole mean residence time; SD: standard deviation; t_1/2_: benznidazole half-life; T_max_: time at which C_max_ occurred.

**TABLE III:** Comparative bioavailability of Abarax^®^ and Benznidazol Lafepe^®^

	C_max_	AUC_0-t_
T/R	97.14	108.22
CI 90%	84.62 - 111.52	82.45 - 142.19
Bioequivalence limit	80.00 - 125.00	80.00 - 125.00
Schuirman test, lower and higher limit	0.014 / 0.004	0.036 / 0.182
Intra-individual variability	19.9%	40.7%

AUC_0-t_: area under curve from zero to 48 h; CI: confidence interval; C_max_: maximum plasma concentration after benznidazole intake; T/R: test drug - reference drug ratio.

**Fig. 2:**
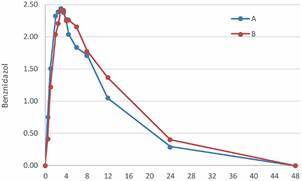
average plasma concentration of benznidazole (mg/mL) relative to intake time (minutes) following administration of Abarax^®^ (A) or Benznidazol Lafepe^®^ (B) obtained with data predicted from the calculated pharmacokinetic constants.

The Fig. 2 shows the average plasma concentration of BNZ (mg/mL) relative to intake time on minutes, following administration of Abarax^®^ and Benznidazol Lafepe^®^. The data predicted from the calculated pharmacokinetic constants presented in Table II were used to build it.

## DISCUSSION

The C_max_ and T_max_ of Abarax^®^ and Benznidazol Lafepe^®^ are within the expected values according to the literature reported at equivalent doses of BNZ. The C_max_ was 3.0 and 2.9 µg/mL for Abarax^®^ and Benznidazol Lafepe^®^, respectively, while this value is reported at 2.2 for Abarax^®(^ ^19^ and 2.9 µg/mL for meta-analysis in the existing literature.^23^ The T_max_ was 3.2 and 2.5 h for Abarax^®^ and Benznidazol Lafepe^®^, respectively, while this value is reported at 3.5 for Abarax^®(^ ^19^ and 2.9 h for meta-analysis in the existing literature.^23^ Finally, the AUC_0-t_ and AUC_0-∞_ and t_1/2_ are values that are below those reported in the literature for both studied formulations. The AUC_0-t_ was 32.0 and 37.5 µg·h/mL for Abarax^®^ and Benznidazol Lafepe^®^, respectively, while this value is reported at 46.4 for Abarax^®(^ ^19^ and 51.3 µg·h/mL for meta-analysis.^23^ The t_1/2_ was 8.0 and 9.1 h for Abarax^®^ and Benznidazol Lafepe^®^, respectively, while this value is reported at 12.1 for Abarax^®(^ ^19^ and 13.3 h for meta-analysis.^23^

It should be noted that the mean difference in the AUC was 10%. However, the large intra-individual variability observed culminated in a CI exceeding the maximum value. The observed intra-individual variability was more than 30%, which is significantly high, so that BNZ can be considered a drug with high variability, compatible with what has been observed by other authors. In the analysis of individual pharmacokinetics, one patient was observed with an atypical behaviour of the curve obtained for the administration of Abarax^®^, expressing a half-life value of 168.28 min. Applying Gruber’s test, this patient is discarded for the pharmacokinetic analysis and calculation of the respective parameters.

Our results suggest also an erratic absorption and/or elimination of the compound, which is manifested by the presence, in many cases, of two or even three concentration peaks in the individual concentration-time curves. This is most likely one of the causes of the large intra-individual variability observed.

As a result of the analysis without the outlier patient, the t_½_ of the two formulations were 9.1 ± 3.6 h BNZ Lafepe^®^ and 8.0 ± 3.5 h for Abarax^®^, with a significance level of p < 0.01, obtained by Wilcoxon paired test. Although these values are statistically different, the observed high inter-individual variability and the large overlap of the 95% CI for t_½_ parameter make that, under pharmacological criteria, both formulations are similar.

To sum up, in this study it was observed that at equivalent doses of BNZ, the C_max_ and T_max_ of Abarax^®^ and Benznidazol Lafepe^®^ were within the expected values according to the literature. Also, the AUC_0-t_, AUC_0-∞_ and t_1/2_ values were below those reported in the literature for both studied formulations. With regard to AUC it cannot be excluded that the differences are due to a “period” effect rather than a real medication difference. This effect could, for example, occur when a drug self-induces/inhibits its metabolism. Visual examination suggests that a carry-over effect would be unlikely, although the absence of the “sequence” factor does not rule it out.

Notably, the mean difference in the AUC was lower than 10%, but exceeding the 90% CI for the higher bioequivalence limit. However, the large intra-individual variability observed culminated in a CI exceeding the maximum value, leading to the recommendation to incorporate more measurement points between 12-48 h in future studies. Finally, although there is a general consensus regarding the values of the main BNZ pharmacokinetic parameters, irrespective of its solid formulations, there is still a lack of data regarding the factors that substantially influence intra-individual variability.
